# Hydro­nium (3-oxo-1-phosphono-1,3-dihydro­isobenzofuran-1-yl)phospho­nate

**DOI:** 10.1107/S1600536809000907

**Published:** 2009-01-10

**Authors:** Carole Barbey, Pascal Retailleau, Erwann Guénin, Nathalie Dupont

**Affiliations:** a Laboratoire de Biophysique Moléculaire Cellulaire et Tissulaire, UMR 7033 CNRS, UFR–SMBH Université Paris-Nord, 74 rue M. Cachin, 93017 Bobigny Cedex, France; b Service de Cristallochimie, Institut de Chimie des Substances Naturelles, CNRS, 1 Av. de la Terrasse, 91198 Gif sur-Yvette Cedex, France

## Abstract

In the title compound, H_3_O^+^·C_8_H_7_O_8_P_2_^−^, the anions form inversion dimmers by way of pairs of O—H⋯O hydrogen bonds involving the phospho­nic functions and *via* the hydro­nium cation. Further O—H⋯O links involving the hydronium cation play a prominant part in the cohesion of the crystal structure by building bridges between bis­phospho­nate pairs, forming infinite ribbons along the *b*-axis direction and by cross-linking these ribbons perpendicularly along the *a*-axis direction, forming an infinite three-dimensional hydrogen-bond network. The benzene ring and the C=O atoms of the furan ring are disordered over two sets of positions of equal occupancy.

## Related literature

For the pharmacological applications of bis­phospho­nates, see Heymann *et al.* (2004 ▶); Rodan & Martin (2000 ▶); Fournier *et al.* (2002 ▶); Hamma-Kourbali *et al.* (2003 ▶); Wood *et al.* (2002 ▶); Martin *et al.* (2001 ▶, 2002 ▶); Sanders *et al.* (2003 ▶). For general background, see Lecouvey *et al.* (2003*a* ▶,*b* ▶); Monteil *et al.* (2005 ▶); Guénin *et al.* (2004 ▶); Lecouvey & Leroux (2000 ▶); Vachal *et al.* (2006 ▶). For related structures, see Sylvestre *et al.* (2001 ▶); Lecouvey *et al.* (2002 ▶).
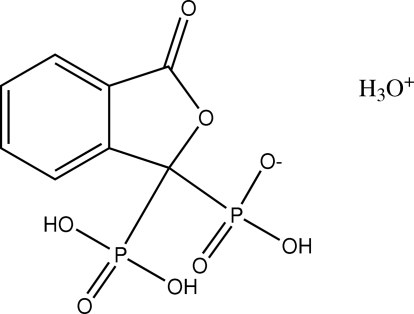


## Experimental

### 

#### Crystal data

H_3_O^+^·C_8_H_7_O_8_P_2_^−^
*M* *_r_* = 312.10
Monoclinic, 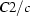

*a* = 26.2271 (9) Å
*b* = 7.2913 (3) Å
*c* = 15.2621 (6) Å
β = 124.103 (2)°
*V* = 2416.66 (16) Å^3^
*Z* = 8
Mo *K*α radiation
μ = 0.40 mm^−1^
*T* = 293 (2) K
0.30 × 0.10 × 0.10 mm

#### Data collection

Nonius KappaCCD diffractometer
Absorption correction: none
14205 measured reflections
2139 independent reflections
1627 reflections with *I* > 2σ(*I*)
*R*_int_ = 0.071

#### Refinement

*R*[*F* ^2^ > 2σ(*F* ^2^)] = 0.045
*wR*(*F* ^2^) = 0.126
*S* = 1.05
2139 reflections
229 parameters
21 restraints
H-atom parameters constrained
Δρ_max_ = 0.33 e Å^−3^
Δρ_min_ = −0.37 e Å^−3^

### 

Data collection: *COLLECT* (Hooft, 1998 ▶); cell refinement: *HKL* (Otwinowski & Minor, 1997 ▶); data reduction: *HKL*; program(s) used to solve structure: *SHELXS97* (Sheldrick, 2008 ▶); program(s) used to refine structure: *SHELXL97* (Sheldrick, 2008 ▶); molecular graphics: *ORTEP-3 for Windows* (Farrugia, 1997 ▶); software used to prepare material for publication: *WinGX* (Farrugia, 1999 ▶) and *CrystalBuilder* (Welter, 2006 ▶).

## Supplementary Material

Crystal structure: contains datablocks global, I. DOI: 10.1107/S1600536809000907/dn2420sup1.cif

Structure factors: contains datablocks I. DOI: 10.1107/S1600536809000907/dn2420Isup2.hkl

Additional supplementary materials:  crystallographic information; 3D view; checkCIF report

## Figures and Tables

**Table 1:** Hydrogen-bond geometry (Å, °)

*D*—H⋯*A*	*D*—H	H⋯*A*	*D*⋯*A*	*D*—H⋯*A*
O11—H11⋯O23^i^	0.82	1.69	2.504 (3)	168
O12—H12⋯O22^ii^	0.82	1.64	2.438 (3)	164
O21—H21⋯O13^iii^	0.82	1.72	2.522 (3)	167
O1*W*—H1*W*⋯O13	0.94	2.09	2.996 (4)	162
O1*W*—H2*W*⋯O23^iv^	0.95	1.90	2.845 (4)	174
O1*W*—H3*W*⋯O2*B*^v^	0.94	1.93	2.875 (10)	177
O1*W*—H3*W*⋯O2*A*^v^	0.94	1.95	2.853 (9)	159

Symmetry codes: (i) 
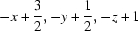
; (ii) 
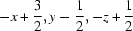
; (iii) 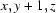; (iv) 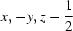; (v) 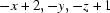.

**Fig. 1.**
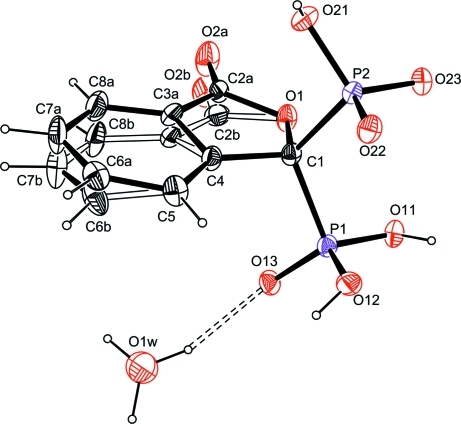
Molecular View of the title compound. Displacement ellipsoids are drawn at the 30% probability level. H atoms are represented as small spheres of arbitrary radii.

## supplementary crystallographic information

### Comment

The title compound, C_8_H_8_O_8_P_2_, belongs to the bisphosphonate family (or 1-hydroxymethylene-1,1-bisphosphonic acids or HMBPs). These compounds are synthetic structural analogues of pyrophosphate and are characterized by an enzymatically stable P—C—P group instead of the P—O—P. They are known to have a wide range of applications. They are clinically used in treatement of various bone diseases, such as Pagets disease, osteoporosis, tumor osteolysis or hypercalcemia of malignancy (Heymann *et al.*, 2004; Rodan & Martin, 2000). They are known to induce inhibition of breast and prostate cancer cell proliferation and more recently to inhibit angiogenesis *in vitro* and *in vivo* (Fournier *et al.*, 2002; Hamma-Kourbali *et al.*, 2003; Wood *et al.*, 2002). In addition, HMBPs have also activity against several trypanosomatid and apicomplexan parasites (Martin *et al.*, 2001; Martin *et al.*, 2002; Sanders *et al.*, 2003). HMBPs are usually obtained from two different synthetic methods (Lecouvey & Leroux, 2000). Unfortunately, these methods are not always suitable for fragile, aromatic or functionalized substrates. Recently we developed a new method of HMBP synthesis from silylated phosphite and acid chlorides (Lecouvey *et al.*, 2003*a*,*b*; Monteil *et al.*, 2005) (or acid anhydrides (Guénin *et al.*, 2004)) that gave an easy access to the obtaining of aromatic and functionalized HMBPs. Using phthalic anhydride as a substrate, a new and original cyclic bisphosphonate was described (Guénin *et al.*, 2004). The cyclic structure of this compound was provided indirectly by IR measurements and further opening of the cycle in basic media. Here we undoubtly proved this cyclic structure, the hydroxy function being part of a lactone. This compound presents a real biological interest as it could act as a prodrug. The hydroxy function which is essential to the HMBP biological properties is in this particular case totally hidden, but could be reformed in the cell by esterase activity. Such acyloxymethyl bis(phosphonate) prodrugs have already been described and the protection shown to be reversible (Vachal *et al.*, 2006).

Bisphosphonate are compounds with super-acid properties, and they easily crystallize as mono salts of sodium or potassium (Sylvestre *et al.*, 2001) or as well characterized solvates (Lecouvey *et al.*, 2002) where crystals generally include water.

The asymetric unit of the title compound is built up from one deprotonated HMBP anion and a H_3_O^+^ cation (Fig. 1) which are linked through OW—H···O hydrogen bonds (Table 1). The crystal structure consists of hydrophilic layers that enclose the hydronium cation and bisphosphonate function where molecules linked by pair and less hydrophilic layers made of aromatic rings attached to the cyclic bisphosphonate structure.

### Experimental

Synthesis of (3-Oxo-1-phosphono-1,3-dihydro-isobenzofuran-1-yl) -phosphonic acid] was done according to the published procedure (Guénin *et al.*, 2004, compound 3 h). Briefly two equivalents of tris(trimethylsilyl)phosphite were added under N_2_ to phtalic anhydride in freshly distilled THF at room temperature. The resulting mixture was then heated at 50°C for 12 h. After evaopration of volatile fractions methanol was added to the residue. After 1 h stirring and methanol evaporation the title compound was washed several times with dimethyl ether. Crystallization was done by slow evaporation at room temperature from a concentrated methanol/ water (9/1) solution to give colorless crystal with max. size 0.3 mm, suitable for diffraction.

### Refinement

All H atoms attached to C or O atoms were fixed geometrically and treated as riding with C—H = 0.93 Å (aromatic) or 0.96 Å (methylene) and O—H = 0.82 Å (hydoxyl) with *U*_iso_(H) = 1.2*U*_eq_(C) (aromatic) and 1.5*U*_eq_(O) for others. Owing to the fact that each of the P2—O22 and P2—O23 bonds seems to be a mixture of single and double bonds and that solvent molecule was 3 times hydrogen donor, the solvent molecule was refined as H_3_O^+^ and the bisphoshonate as the basic form. H atoms of the hydronium were located in difference Fourier syntheses and initially refined using restraints (O-H= 0.93 (1)Å) with *U*_iso_(H) = 1.5*U*_eq_(O). In the last stage of refinement they were treated as riding on the parent O atom.

Disorder of the cyclic structure was modeled with two different positions per disordered atom with occupation factors of 0.5. The two disordered part were refined using the tools, PART and SAME,  available in SHELXL-97 (Sheldrick, 2008).

### Crystal data

**Table:**

H_3_O^+^·C_8_H_7_O_8_P_2_^−^	*F*(000) = 1280
*M**_r_* = 312.10	*D*_x_ = 1.716 Mg m^−^^3^
Monoclinic, *C*2/*c*	Mo *K*α radiation, λ = 0.71069 Å
Hall symbol: -C 2yc	Cell parameters from 2338 reflections
*a* = 26.2271 (9) Å	θ = 0.4–25.4°
*b* = 7.2913 (3) Å	µ = 0.40 mm^−^^1^
*c* = 15.2621 (6) Å	*T* = 293 K
β = 124.103 (2)°	Parallelepipedic, colourless
*V* = 2416.66 (16) Å^3^	0.30 × 0.10 × 0.10 mm
*Z* = 8	

### Data collection

**Table:**

Nonius KappaCCD diffractometer	1627 reflections with *I* > 2σ(*I*)
Radiation source: fine-focus sealed tube	*R*_int_ = 0.071
graphite	θ_max_ = 25.4°, θ_min_ = 3.0°
Detector resolution: 9 pixels mm^-1^	*h* = −31→30
φ and ω scans	*k* = −8→8
14205 measured reflections	*l* = −18→17
2139 independent reflections	

### Refinement

**Table:**

Refinement on *F*^2^	Primary atom site location: structure-invariant direct methods
Least-squares matrix: full	Secondary atom site location: difference Fourier map
*R*[*F*^2^ > 2σ(*F*^2^)] = 0.045	Hydrogen site location: inferred from neighbouring sites
*wR*(*F*^2^) = 0.126	H-atom parameters constrained
*S* = 1.05	*w* = 1/[σ^2^(*F*_o_^2^) + (0.0625*P*)^2^ + 4.0148*P*] where *P* = (*F*_o_^2^ + 2*F*_c_^2^)/3
2139 reflections	(Δ/σ)_max_ = 0.001
229 parameters	Δρ_max_ = 0.33 e Å^−^^3^
21 restraints	Δρ_min_ = −0.37 e Å^−^^3^

### Special details

**Table:**

Geometry. All esds (except the esd in the dihedral angle between two l.s. planes) are estimated using the full covariance matrix. The cell esds are taken into account individually in the estimation of esds in distances, angles and torsion angles; correlations between esds in cell parameters are only used when they are defined by crystal symmetry. An approximate (isotropic) treatment of cell esds is used for estimating esds involving l.s. planes.
Refinement. Refinement of *F*^2^ against ALL reflections. The weighted *R*-factor *wR* and goodness of fit *S* are based on *F*^2^, conventional *R*-factors *R* are based on *F*, with *F* set to zero for negative *F*^2^. The threshold expression of *F*^2^ > σ(*F*^2^) is used only for calculating *R*-factors(gt) *etc*. and is not relevant to the choice of reflections for refinement. *R*-factors based on *F*^2^ are statistically about twice as large as those based on *F*, and *R*- factors based on ALL data will be even larger.

### Fractional atomic coordinates and isotropic or equivalent isotropic displacement parameters (Å^2^)

**Table:**

	*x*	*y*	*z*	*U*_iso_*/*U*_eq_	Occ. (<1)
P1	0.81092 (4)	0.03803 (12)	0.41201 (6)	0.0268 (3)	
O11	0.79680 (10)	−0.0117 (3)	0.49420 (18)	0.0338 (6)	
H11	0.7615	0.0191	0.4721	0.051*	
O12	0.75192 (9)	0.0932 (3)	0.30772 (17)	0.0338 (6)	
H12	0.7528	0.0541	0.2582	0.051*	
O13	0.84830 (10)	−0.1086 (3)	0.40406 (18)	0.0339 (6)	
P2	0.82143 (3)	0.45428 (11)	0.46825 (6)	0.0250 (3)	
O23	0.80619 (9)	0.4158 (3)	0.54732 (17)	0.0316 (5)	
O22	0.76507 (9)	0.5004 (3)	0.36019 (17)	0.0323 (6)	
O21	0.87289 (10)	0.5997 (3)	0.50941 (18)	0.0339 (6)	
H21	0.8605	0.6856	0.4676	0.051*	
C1	0.85893 (13)	0.2462 (4)	0.4606 (2)	0.0252 (7)	
O1	0.90991 (9)	0.2067 (3)	0.57008 (15)	0.0329 (6)	
C2A	0.9660 (4)	0.2436 (17)	0.5830 (7)	0.030 (3)	0.50
O2A	1.0134 (4)	0.2317 (14)	0.6679 (7)	0.058 (3)	0.50
C3A	0.9536 (4)	0.2866 (17)	0.4802 (7)	0.032 (3)	0.50
C4	0.89030 (14)	0.2748 (5)	0.4041 (2)	0.0295 (7)	
C5	0.86705 (16)	0.3115 (5)	0.2986 (3)	0.0373 (8)	
H5	0.8250	0.3067	0.2467	0.045*	
C6A	0.9093 (5)	0.356 (2)	0.2737 (11)	0.037 (4)	0.50
H6A	0.8947	0.3890	0.2046	0.045*	0.50
C7A	0.9732 (6)	0.3515 (19)	0.3495 (10)	0.056 (4)	0.50
H7A	1.0003	0.3736	0.3296	0.068*	0.50
C8A	0.9952 (5)	0.3150 (15)	0.4523 (9)	0.050 (3)	0.50
H8A	1.0374	0.3092	0.5032	0.060*	0.50
C2B	0.9639 (5)	0.1751 (16)	0.5743 (9)	0.038 (4)	0.50
O2B	1.0105 (4)	0.1345 (12)	0.6576 (8)	0.053 (2)	0.50
C3B	0.9517 (4)	0.2220 (17)	0.4728 (8)	0.030 (3)	0.50
C6B	0.9075 (6)	0.297 (3)	0.2668 (12)	0.045 (5)	0.50
H6B	0.8922	0.3094	0.1955	0.055*	0.50
C7B	0.9703 (6)	0.264 (2)	0.3393 (11)	0.062 (5)	0.50
H7B	0.9971	0.2723	0.3177	0.074*	0.50
C8B	0.9926 (5)	0.2211 (16)	0.4419 (10)	0.052 (3)	0.50
H8B	1.0339	0.1918	0.4897	0.062*	0.50
O1W	0.85750 (13)	−0.1636 (4)	0.2188 (2)	0.0646 (9)	
H1W	0.8473	−0.1639	0.2690	0.097*	
H2W	0.8410	−0.2545	0.1654	0.097*	
H3W	0.9009	−0.1544	0.2575	0.097*	

### Atomic displacement parameters (Å^2^)

**Table:**

	*U*^11^	*U*^22^	*U*^33^	*U*^12^	*U*^13^	*U*^23^
P1	0.0238 (4)	0.0260 (5)	0.0315 (5)	0.0011 (3)	0.0160 (4)	0.0003 (3)
O11	0.0267 (12)	0.0395 (14)	0.0395 (13)	0.0046 (10)	0.0212 (11)	0.0068 (11)
O12	0.0250 (11)	0.0408 (14)	0.0277 (11)	0.0002 (10)	0.0099 (10)	−0.0051 (10)
O13	0.0351 (12)	0.0286 (13)	0.0461 (14)	0.0040 (10)	0.0277 (12)	−0.0010 (10)
P2	0.0219 (4)	0.0269 (5)	0.0268 (4)	−0.0013 (3)	0.0141 (4)	0.0004 (3)
O23	0.0298 (11)	0.0380 (13)	0.0329 (12)	0.0034 (10)	0.0211 (11)	0.0044 (10)
O22	0.0224 (11)	0.0385 (14)	0.0311 (12)	0.0041 (10)	0.0120 (10)	0.0051 (10)
O21	0.0259 (11)	0.0296 (13)	0.0391 (13)	−0.0050 (10)	0.0138 (11)	0.0014 (10)
C1	0.0174 (14)	0.0338 (18)	0.0203 (14)	0.0011 (13)	0.0081 (13)	0.0012 (12)
O1	0.0210 (11)	0.0494 (15)	0.0257 (11)	0.0043 (10)	0.0116 (10)	0.0059 (10)
C2A	0.012 (4)	0.046 (9)	0.026 (5)	0.000 (4)	0.007 (4)	−0.008 (4)
O2A	0.022 (4)	0.111 (8)	0.032 (4)	−0.004 (5)	0.009 (3)	−0.001 (5)
C3A	0.024 (4)	0.033 (9)	0.038 (5)	−0.008 (4)	0.016 (4)	−0.005 (4)
C4	0.0237 (16)	0.0341 (19)	0.0338 (16)	0.0004 (14)	0.0180 (15)	0.0002 (14)
C5	0.0329 (18)	0.046 (2)	0.0331 (18)	0.0081 (16)	0.0186 (16)	0.0073 (16)
C6A	0.046 (6)	0.032 (11)	0.039 (5)	0.008 (5)	0.027 (5)	0.006 (5)
C7A	0.050 (6)	0.086 (12)	0.057 (6)	−0.004 (7)	0.045 (6)	0.005 (7)
C8A	0.032 (5)	0.074 (9)	0.048 (5)	−0.016 (6)	0.026 (4)	−0.010 (6)
C2B	0.033 (6)	0.035 (8)	0.043 (6)	−0.005 (4)	0.019 (5)	−0.010 (5)
O2B	0.020 (3)	0.083 (7)	0.038 (4)	0.008 (4)	0.005 (3)	−0.002 (5)
C3B	0.024 (4)	0.028 (8)	0.039 (5)	−0.006 (4)	0.019 (4)	−0.009 (4)
C6B	0.073 (8)	0.036 (12)	0.053 (7)	0.017 (6)	0.051 (7)	0.014 (6)
C7B	0.050 (7)	0.091 (12)	0.070 (8)	0.011 (7)	0.049 (7)	0.024 (8)
C8B	0.029 (5)	0.074 (9)	0.061 (6)	−0.001 (6)	0.030 (5)	0.007 (7)
O1W	0.0470 (16)	0.081 (2)	0.0599 (18)	0.0001 (16)	0.0266 (15)	−0.0147 (16)

### Geometric parameters (Å, °)

**Table:**

P1—O13	1.501 (2)	C4—C3B	1.395 (9)
P1—O12	1.526 (2)	C5—C6A	1.397 (11)
P1—O11	1.537 (2)	C5—C6B	1.397 (11)
P1—C1	1.842 (3)	C5—H5	0.9300
O11—H11	0.8200	C6A—C7A	1.405 (11)
O12—H12	0.8200	C6A—H6A	0.9300
P2—O23	1.495 (2)	C7A—C8A	1.360 (11)
P2—O22	1.511 (2)	C7A—H7A	0.9300
P2—O21	1.546 (2)	C8A—H8A	0.9300
P2—C1	1.847 (3)	C2B—O2B	1.207 (10)
O21—H21	0.8200	C2B—C3B	1.437 (11)
C1—O1	1.469 (3)	C3B—C8B	1.393 (10)
C1—C4	1.503 (4)	C6B—C7B	1.396 (12)
O1—C2A	1.397 (9)	C6B—H6B	0.9300
O1—C2B	1.399 (10)	C7B—C8B	1.366 (11)
C2A—O2A	1.193 (9)	C7B—H7B	0.9300
C2A—C3A	1.447 (10)	C8B—H8B	0.9300
C3A—C8A	1.390 (10)	O1W—H1W	0.9423
C3A—C4	1.397 (9)	O1W—H2W	0.9469
C4—C5	1.391 (4)	O1W—H3W	0.9450
			
O13—P1—O12	115.47 (13)	C3A—C4—C1	108.0 (5)
O13—P1—O11	111.42 (13)	C4—C5—C6A	117.4 (6)
O12—P1—O11	110.24 (12)	C4—C5—C6B	117.6 (7)
O13—P1—C1	106.83 (13)	C6A—C5—C6B	18.0 (14)
O12—P1—C1	105.50 (13)	C4—C5—H5	121.3
O11—P1—C1	106.80 (13)	C6A—C5—H5	121.3
P1—O11—H11	109.5	C6B—C5—H5	118.0
P1—O12—H12	109.5	C5—C6A—C7A	122.2 (10)
O23—P2—O22	112.47 (12)	C5—C6A—H6A	118.9
O23—P2—O21	111.60 (13)	C7A—C6A—H6A	118.9
O22—P2—O21	112.81 (13)	C8A—C7A—C6A	119.5 (11)
O23—P2—C1	106.84 (13)	C8A—C7A—H7A	120.3
O22—P2—C1	110.08 (13)	C6A—C7A—H7A	120.3
O21—P2—C1	102.39 (13)	C7A—C8A—C3A	119.0 (10)
P2—O21—H21	109.5	C7A—C8A—H8A	120.5
O1—C1—C4	103.8 (2)	C3A—C8A—H8A	120.5
O1—C1—P1	106.1 (2)	O2B—C2B—O1	119.4 (10)
C4—C1—P1	110.7 (2)	O2B—C2B—C3B	132.5 (10)
O1—C1—P2	105.48 (18)	O1—C2B—C3B	107.6 (8)
C4—C1—P2	113.9 (2)	C8B—C3B—C4	122.0 (8)
P1—C1—P2	115.74 (15)	C8B—C3B—C2B	128.0 (9)
C2A—O1—C2B	21.1 (7)	C4—C3B—C2B	110.0 (7)
C2A—O1—C1	109.7 (4)	C7B—C6B—C5	121.7 (11)
C2B—O1—C1	109.7 (5)	C7B—C6B—H6B	119.2
O2A—C2A—O1	120.8 (9)	C5—C6B—H6B	119.2
O2A—C2A—C3A	131.1 (9)	C8B—C7B—C6B	120.3 (11)
O1—C2A—C3A	108.1 (7)	C8B—C7B—H7B	119.9
C8A—C3A—C4	121.7 (8)	C6B—C7B—H7B	119.9
C8A—C3A—C2A	128.8 (9)	C7B—C8B—C3B	118.1 (10)
C4—C3A—C2A	109.1 (7)	C7B—C8B—H8B	120.9
C5—C4—C3B	119.7 (5)	C3B—C8B—H8B	120.9
C5—C4—C3A	119.7 (5)	H1W—O1W—H2W	119.8
C3B—C4—C3A	19.8 (8)	H1W—O1W—H3W	106.4
C5—C4—C1	131.7 (3)	H2W—O1W—H3W	113.4
C3B—C4—C1	107.6 (5)		

### Hydrogen-bond geometry (Å, °)

**Table:**

*D*—H···*A*	*D*—H	H···*A*	*D*···*A*	*D*—H···*A*
O11—H11···O23^i^	0.82	1.69	2.504 (3)	168
O12—H12···O22^ii^	0.82	1.64	2.438 (3)	164
O21—H21···O13^iii^	0.82	1.72	2.522 (3)	167
O1W—H1W···O13	0.94	2.09	2.996 (4)	162
O1W—H2W···O23^iv^	0.95	1.90	2.845 (4)	174
O1W—H3W···O2B^v^	0.94	1.93	2.875 (10)	177
O1W—H3W···O2A^v^	0.94	1.95	2.853 (9)	159

Symmetry codes: (i) −*x*+3/2, −*y*+1/2, −*z*+1; (ii) −*x*+3/2, *y*−1/2, −*z*+1/2; (iii) *x*, *y*+1, *z*; (iv) *x*, −*y*, *z*−1/2; (v) −*x*+2, −*y*, −*z*+1.

## References

[bb1] Farrugia, L. J. (1997). *J. Appl. Cryst.* **30**, 565.

[bb2] Farrugia, L. J. (1999). *J. Appl. Cryst.* **32**, 837–838.

[bb3] Fournier, P., Boissier, S., Filleur, S., Guglielmi, J., Cabon, F., Colombel, M. & Clezardin, P. (2002). *Cancer Res.* **62**, 6538-6544. 12438248

[bb4] Guénin, E., Degache, E., Liquier, J. & Lecouvey, M. (2004). *Eur. J. Org. Chem.* pp. 2983–2987.

[bb5] Hamma-Kourbali, Y., Di Benedetto, M., Ledoux, D., Oudar, O., Leroux, Y., Lecouvey, M. & Kraemer, M. (2003). *Biochem. Biophys. Res. Commun.* **310**, 816–823. 10.1016/j.bbrc.2003.09.083 14550277

[bb6] Heymann, D., Ory, B., Gouin, F., Green, J. R. & Redini, F. (2004). *Trends Mol. Med.* **10** 337–343. 10.1016/j.molmed.2004.05.007 15242682

[bb7] Hooft, R. W. W. (1998). *COLLECT* Nonius BV, Delft, The Netherlands.

[bb8] Lecouvey, M., Barbey, C., Navaza, A., Neuman, A. & Prangé, T. (2002). *Acta Cryst.* C**58**, o521–o524. 10.1107/s0108270102012386 12154318

[bb9] Lecouvey, M. & Leroux, Y. (2000). *Heteroat. Chem* **11**, 556–561.

[bb10] Lecouvey, M., Leroux, Y., Kraemer, M., Crepin, M., el Manouni, D., Louriki, M. (2003*a*). *Chem. Abstr* **138**, 122736.

[bb11] Lecouvey, M., Leroux, Y., Kraemer, M., Crepin, M., el Manouni, D., Louriki, M. (2003*b*). World Patent WO 03/008425.

[bb12] Martin, M. B., Sanders, J. M., Kendrick, H., de Luca-Fradley, K., Lewis, J. C., Grimley, J. S., Van Brussel, E. M., Olsen, J. R., Meints, G. A., Burzynska, A., Kafarski, P., Croft, S. L. & Oldfield, E. (2002). *J. Med. Chem.* **45**, 2904–2914. 10.1021/jm0102809 12086478

[bb13] Martin, M. B., Grimley, J. S., Lewis, J. C., Heath, H. T., 3rd, Bailey, B. N., Kendrick, H., Yardley, V., Caldera, A., Lira, R., Urbina, J. A., Moreno, S. N., Docampo, R., Croft, S. L., Oldfield, E. (2001). *J. Med. Chem.* **44**, 909–916. 10.1021/jm0002578 11300872

[bb14] Monteil, M., Guenin, E., Migianu, E., Lutomski, D. & Lecouvey, M. (2005). *Tetrahedron*, **61**, 7528–7537.

[bb15] Otwinowski, Z. & Minor, W. (1997). *Methods in Enzymology*, Vol. 276, *Macromolecular Crystallography*, Part A, edited by C. W. Carter Jr & R. M. Sweet, pp. 307–326. New York: Academic Press.

[bb16] Rodan, G. A. & Martin, T. J. (2000). *Science*, **289**, 1508–1514. 10.1126/science.289.5484.1508 10968781

[bb17] Sanders, J. M., Gomez, A. O., Mao, J., Meints, G. A., Van Brussel, E. M., Burzynska, A., Kafarski, P., Gonzalez-Pacanowska, D. & Oldfield, E. (2003). *J. Med. Chem.* **46**, 5171–5183. 10.1021/jm0302344 14613320

[bb18] Sheldrick, G. M. (2008). *Acta Cryst.* A**64**, 112–122. 10.1107/S0108767307043930 18156677

[bb19] Sylvestre, J. P., Nguyen, Q. D. & Leroux, Y. (2001). *Heteroat. Chem.* **12**, 73–90.

[bb20] Vachal, P., Hale, J. J., Lu, Z., Streckfuss, E. C., Mills, S. G., MacCoss, M., Yin, D. H., Algayer, K., Manser, K., Kesioglou, F., Ghosh, S. & Alani, L. L. (2006). *J. Med. Chem* **49**, 3060–3063. 10.1021/jm060398v 16722624

[bb21] Welter, R. (2006). *Acta Cryst.* A**62**, s252.

[bb22] Wood, J., Bonjean, K., Ruetz, S., Bellahcene, A., Devy, L., Foidart, J. M., Castronovo, V. & Green, J. R. (2002). *J. Pharmacol. Exp. Ther.* **302**, 1055–1061. 10.1124/jpet.102.035295 12183663

